# 1-(1-Benzofuran-2-yl)-2-(phenyl­sulfon­yl)ethanone

**DOI:** 10.1107/S1600536811037366

**Published:** 2011-09-17

**Authors:** Hatem A. Abdel-Aziz, Seik Weng Ng, Edward R. T. Tiekink

**Affiliations:** a Department of Pharmaceutical Chemistry, College of Pharmacy, King Saud University, Riyadh 11451, Saudi Arabia; b Department of Chemistry, University of Malaya, 50603 Kuala Lumpur, Malaysia; c Chemistry Department, Faculty of Science, King Abdulaziz University, PO Box 80203 Jeddah, Saudi Arabia

## Abstract

The overall mol­ecular conformation of the title compound, C_16_H_12_O_4_S, is elongated, the dihedral angle formed between the benzofuran (r.m.s. deviation = 0.018 Å) and benzene rings being 24.81 (6)°. Both sulfonyl O atoms lie to one side of the S-bound benzene ring, and the carbonyl and furan O atoms are *syn* to each other. Supra­molecular arrays parallel to (101) sustained by C—H⋯O contacts feature in the crystal packing.

## Related literature

For the biological activity of sulfones, see: Garuti *et al.* (2002 ▶), and of benzofuran, see: Abdel-Aziz & Mekawey (2009 ▶). For previous work on the chemistry and biological activity of benzofurans, see: Abdel-Wahab *et al.* (2009 ▶); Abdel-Aziz *et al.* (2009 ▶, 2011 ▶). For the synthesis, see: Takahashi *et al.* (1986 ▶).
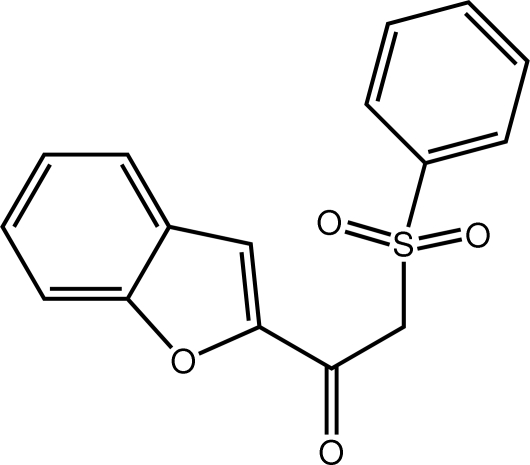


## Experimental

### 

#### Crystal data

C_16_H_12_O_4_S
*M* *_r_* = 300.32
Monoclinic, 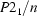

*a* = 10.7560 (2) Å
*b* = 4.7855 (1) Å
*c* = 26.1838 (5) Å
β = 91.024 (2)°
*V* = 1347.54 (5) Å^3^
*Z* = 4
Cu *K*α radiation
μ = 2.27 mm^−1^
*T* = 100 K
0.35 × 0.15 × 0.15 mm

#### Data collection

Agilent SuperNova Dual diffractometer with an Atlas detector
Absorption correction: multi-scan (*CrysAlis PRO*; Agilent, 2010 ▶) *T*_min_ = 0.598, *T*_max_ = 1.000
4617 measured reflections
2650 independent reflections
2495 reflections with *I* > 2σ(*I*)
*R*_int_ = 0.016

#### Refinement

*R*[*F* ^2^ > 2σ(*F* ^2^)] = 0.032
*wR*(*F* ^2^) = 0.086
*S* = 1.04
2650 reflections
190 parameters
H-atom parameters constrained
Δρ_max_ = 0.34 e Å^−3^
Δρ_min_ = −0.47 e Å^−3^

### 

Data collection: *CrysAlis PRO* (Agilent, 2010 ▶); cell refinement: *CrysAlis PRO*; data reduction: *CrysAlis PRO*; program(s) used to solve structure: *SHELXS97* (Sheldrick, 2008 ▶); program(s) used to refine structure: *SHELXL97* (Sheldrick, 2008 ▶); molecular graphics: *ORTEP-3* (Farrugia, 1997 ▶) and *DIAMOND* (Brandenburg, 2006 ▶); software used to prepare material for publication: *publCIF* (Westrip, 2010 ▶).

## Supplementary Material

Crystal structure: contains datablock(s) global, I. DOI: 10.1107/S1600536811037366/pk2346sup1.cif

Structure factors: contains datablock(s) I. DOI: 10.1107/S1600536811037366/pk2346Isup2.hkl

Supplementary material file. DOI: 10.1107/S1600536811037366/pk2346Isup3.cml

Additional supplementary materials:  crystallographic information; 3D view; checkCIF report

## Figures and Tables

**Table 1:** Hydrogen-bond geometry (Å, °)

*D*—H⋯*A*	*D*—H	H⋯*A*	*D*⋯*A*	*D*—H⋯*A*
C3—H3⋯O3^i^	0.95	2.55	3.1808 (19)	124
C7—H7a⋯O2^ii^	0.99	2.57	3.5383 (17)	165
C7—H7b⋯O1^i^	0.99	2.47	3.3746 (17)	152
C15—H15⋯O3^iii^	0.95	2.47	3.2742 (17)	142

Symmetry codes: (i) 
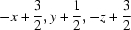
; (ii) 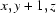; (iii) 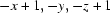.

**Fig. 1.**
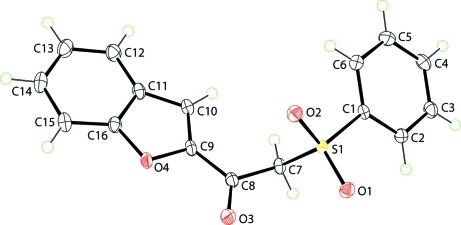
The molecular structure of (I) showing displacement ellipsoids at the 50% probability level.

**Fig. 2.**
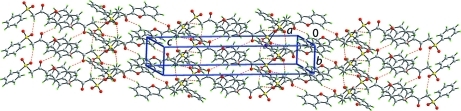
A view of the supramolecular array parallel to (101) in (I) mediated by C—H···O contacts shown as orange dashed lines.

**Fig. 3.**
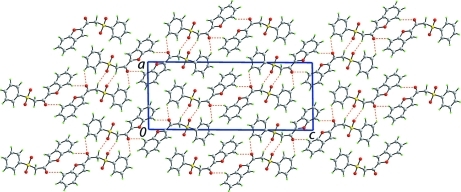
A view in projection down the b axis of the unit-cell contents of (I) highlighting the stacking of layers; the C—H···O contacts are shown as orange dashed lines.

## supplementary crystallographic information

### Comment

The investigation of the title compound, (I), a composite of sulphone and benzofuran groups, was motivated by the known biological activity of each component (Garuti *et al.*, 2002; Abdel-Aziz & Mekawey, 2009) and represents a continuation of on-going biological and structural studies in this area (Abdel-Wahab *et al.*, 2009; Abdel-Aziz *et al.*, 2009; Abdel-Aziz *et al.*, 2011).

With respect to the S-bound benzene ring in (I), Fig. 1, the two sulfonyl-O atoms lie to one side and the methylene group to the other. The benzofuran group is planar (r.m.s. deviation = 0.018 Å) and is splayed out with respect to the rest of the molecule.  The dihedral angle between the S-bound benzene and benzofuran rings is 24.81 (6) ° so that overall the molecule has a flattened shape.  The carbonyl- and benzofuran-O atoms are *syn* to each other.

The crystal packing is dominated by C—H···O interactions, Table 1, involving all but the benzofuran-O4 atom.  These lead to the formation of supramolecular arrays parallel to (101), Fig. 2. There are no specific interactions between the layers, Fig. 3.

### Experimental

The title compound was prepared according to the reported method (Takahashi *et al.*, 1986).  The yellow crystals were isolated from a mixture of EtOH/DMF (*v*/*v* = 3/1) by slow evaporation at room temperature.

### Refinement

Carbon-bound H-atoms were placed in calculated positions [C—H 0.95 to 0.99 Å, *U*_iso_(H) 1.2*U*_eq_(C)] and were included in the refinement in the riding model approximation.

### Crystal data

**Table:**

C_16_H_12_O_4_S	*F*(000) = 624
*M**_r_* = 300.32	*D*_x_ = 1.480 Mg m^−^^3^
Monoclinic, *P*2_1_/*n*	Cu *K*α radiation, λ = 1.5418 Å
Hall symbol: -P 2yn	Cell parameters from 3182 reflections
*a* = 10.7560 (2) Å	θ = 3.4–74.0°
*b* = 4.7855 (1) Å	µ = 2.27 mm^−^^1^
*c* = 26.1838 (5) Å	*T* = 100 K
β = 91.024 (2)°	Prism, yellow
*V* = 1347.54 (5) Å^3^	0.35 × 0.15 × 0.15 mm
*Z* = 4	

### Data collection

**Table:**

Agilent SuperNova Dual diffractometer with an Atlas detector	2650 independent reflections
Radiation source: SuperNova (Cu) X-ray Source	2495 reflections with *I* > 2σ(*I*)
mirror	*R*_int_ = 0.016
Detector resolution: 10.4 pixels mm^-1^	θ_max_ = 74.2°, θ_min_ = 3.4°
ω scans	*h* = −12→13
Absorption correction: multi-scan (*CrysAlis PRO*; Agilent, 2010)	*k* = −5→3
*T*_min_ = 0.598, *T*_max_ = 1.000	*l* = −31→32
4617 measured reflections	

### Refinement

**Table:**

Refinement on *F*^2^	Primary atom site location: structure-invariant direct methods
Least-squares matrix: full	Secondary atom site location: difference Fourier map
*R*[*F*^2^ > 2σ(*F*^2^)] = 0.032	Hydrogen site location: inferred from neighbouring sites
*wR*(*F*^2^) = 0.086	H-atom parameters constrained
*S* = 1.04	*w* = 1/[σ^2^(*F*_o_^2^) + (0.0498*P*)^2^ + 0.6486*P*] where *P* = (*F*_o_^2^ + 2*F*_c_^2^)/3
2650 reflections	(Δ/σ)_max_ = 0.002
190 parameters	Δρ_max_ = 0.34 e Å^−^^3^
0 restraints	Δρ_min_ = −0.47 e Å^−^^3^

### Special details

**Table:**

Geometry. All e.s.d.'s (except the e.s.d. in the dihedral angle between two l.s. planes) are estimated using the full covariance matrix. The cell e.s.d.'s are taken into account individually in the estimation of e.s.d.'s in distances, angles and torsion angles; correlations between e.s.d.'s in cell parameters are only used when they are defined by crystal symmetry. An approximate (isotropic) treatment of cell e.s.d.'s is used for estimating e.s.d.'s involving l.s. planes.
Refinement. Refinement of *F*^2^ against ALL reflections. The weighted *R*-factor *wR* and goodness of fit *S* are based on *F*^2^, conventional *R*-factors *R* are based on *F*, with *F* set to zero for negative *F*^2^. The threshold expression of *F*^2^ > σ(*F*^2^) is used only for calculating *R*-factors(gt) *etc*. and is not relevant to the choice of reflections for refinement. *R*-factors based on *F*^2^ are statistically about twice as large as those based on *F*, and *R*- factors based on ALL data will be even larger.

### Fractional atomic coordinates and isotropic or equivalent isotropic displacement parameters (Å^2^)

**Table:**

	*x*	*y*	*z*	*U*_iso_*/*U*_eq_	
S1	0.53618 (3)	0.14747 (7)	0.735119 (11)	0.01251 (11)	
O1	0.63911 (9)	−0.0313 (2)	0.74882 (4)	0.0174 (2)	
O2	0.42375 (9)	0.0193 (2)	0.71500 (4)	0.0178 (2)	
O3	0.64086 (10)	0.0614 (2)	0.62454 (4)	0.0228 (2)	
O4	0.46617 (9)	0.2296 (2)	0.55532 (4)	0.0173 (2)	
C1	0.49885 (13)	0.3575 (3)	0.78787 (5)	0.0137 (3)	
C2	0.59334 (13)	0.4305 (3)	0.82217 (5)	0.0163 (3)	
H2	0.6754	0.3613	0.8182	0.020*	
C3	0.56524 (14)	0.6072 (3)	0.86253 (6)	0.0201 (3)	
H3	0.6284	0.6594	0.8865	0.024*	
C4	0.44511 (14)	0.7074 (3)	0.86776 (5)	0.0218 (3)	
H4	0.4266	0.8293	0.8952	0.026*	
C5	0.35163 (14)	0.6309 (3)	0.83320 (6)	0.0225 (3)	
H5	0.2696	0.7000	0.8373	0.027*	
C6	0.37766 (13)	0.4545 (3)	0.79284 (5)	0.0183 (3)	
H6	0.3142	0.4008	0.7691	0.022*	
C7	0.58626 (13)	0.3948 (3)	0.68830 (5)	0.0156 (3)	
H7A	0.5339	0.5648	0.6894	0.019*	
H7B	0.6737	0.4494	0.6953	0.019*	
C8	0.57443 (13)	0.2571 (3)	0.63613 (5)	0.0159 (3)	
C9	0.47739 (13)	0.3641 (3)	0.60172 (5)	0.0152 (3)	
C10	0.39035 (13)	0.5678 (3)	0.60687 (5)	0.0166 (3)	
H10	0.3806	0.6887	0.6353	0.020*	
C11	0.31615 (13)	0.5640 (3)	0.56082 (5)	0.0166 (3)	
C12	0.21147 (14)	0.7095 (3)	0.54235 (6)	0.0218 (3)	
H12	0.1746	0.8536	0.5619	0.026*	
C13	0.16361 (15)	0.6368 (3)	0.49482 (6)	0.0242 (3)	
H13	0.0919	0.7305	0.4818	0.029*	
C14	0.21907 (15)	0.4270 (3)	0.46533 (6)	0.0231 (3)	
H14	0.1843	0.3841	0.4327	0.028*	
C15	0.32250 (15)	0.2813 (3)	0.48237 (5)	0.0205 (3)	
H15	0.3604	0.1402	0.4624	0.025*	
C16	0.36750 (13)	0.3544 (3)	0.53055 (5)	0.0163 (3)	

### Atomic displacement parameters (Å^2^)

**Table:**

	*U*^11^	*U*^22^	*U*^33^	*U*^12^	*U*^13^	*U*^23^
S1	0.01469 (18)	0.01156 (19)	0.01124 (17)	−0.00044 (11)	−0.00085 (12)	−0.00152 (11)
O1	0.0191 (5)	0.0154 (5)	0.0178 (5)	0.0036 (4)	−0.0021 (4)	−0.0012 (4)
O2	0.0177 (5)	0.0180 (5)	0.0176 (5)	−0.0042 (4)	−0.0018 (4)	−0.0033 (4)
O3	0.0220 (5)	0.0289 (6)	0.0174 (5)	0.0066 (5)	−0.0021 (4)	−0.0065 (4)
O4	0.0208 (5)	0.0204 (5)	0.0106 (4)	0.0017 (4)	−0.0017 (4)	−0.0029 (4)
C1	0.0182 (7)	0.0128 (7)	0.0101 (6)	−0.0001 (5)	0.0012 (5)	0.0002 (5)
C2	0.0163 (6)	0.0180 (7)	0.0145 (6)	−0.0002 (5)	−0.0001 (5)	−0.0007 (5)
C3	0.0223 (7)	0.0234 (8)	0.0146 (7)	−0.0032 (6)	−0.0009 (5)	−0.0032 (6)
C4	0.0267 (8)	0.0233 (8)	0.0154 (7)	0.0009 (6)	0.0048 (6)	−0.0052 (6)
C5	0.0193 (7)	0.0267 (8)	0.0215 (7)	0.0046 (6)	0.0030 (6)	−0.0032 (6)
C6	0.0178 (7)	0.0209 (8)	0.0161 (7)	0.0009 (6)	−0.0010 (5)	0.0000 (6)
C7	0.0201 (7)	0.0150 (7)	0.0118 (6)	−0.0028 (5)	−0.0012 (5)	−0.0009 (5)
C8	0.0168 (6)	0.0187 (7)	0.0123 (6)	−0.0034 (5)	0.0007 (5)	−0.0015 (5)
C9	0.0192 (7)	0.0169 (7)	0.0096 (6)	−0.0041 (5)	0.0002 (5)	−0.0013 (5)
C10	0.0221 (7)	0.0159 (7)	0.0117 (6)	−0.0023 (6)	0.0018 (5)	0.0000 (5)
C11	0.0218 (7)	0.0155 (7)	0.0125 (6)	−0.0031 (6)	0.0011 (5)	0.0018 (5)
C12	0.0263 (8)	0.0209 (7)	0.0183 (7)	0.0029 (6)	0.0007 (6)	0.0028 (6)
C13	0.0256 (8)	0.0257 (9)	0.0211 (7)	0.0003 (6)	−0.0047 (6)	0.0076 (6)
C14	0.0307 (8)	0.0235 (8)	0.0149 (7)	−0.0059 (7)	−0.0061 (6)	0.0031 (6)
C15	0.0282 (8)	0.0196 (7)	0.0135 (7)	−0.0024 (6)	−0.0012 (6)	−0.0008 (6)
C16	0.0188 (7)	0.0170 (7)	0.0132 (6)	−0.0028 (5)	−0.0004 (5)	0.0027 (5)

### Geometric parameters (Å, °)

**Table:**

S1—O1	1.4394 (10)	C7—C8	1.5201 (18)
S1—O2	1.4468 (10)	C7—H7A	0.9900
S1—C1	1.7603 (14)	C7—H7B	0.9900
S1—C7	1.7936 (15)	C8—C9	1.460 (2)
O3—C8	1.2193 (18)	C9—C10	1.360 (2)
O4—C16	1.3708 (17)	C10—C11	1.4343 (19)
O4—C9	1.3781 (16)	C10—H10	0.9500
C1—C2	1.3891 (19)	C11—C16	1.398 (2)
C1—C6	1.3919 (19)	C11—C12	1.403 (2)
C2—C3	1.391 (2)	C12—C13	1.383 (2)
C2—H2	0.9500	C12—H12	0.9500
C3—C4	1.387 (2)	C13—C14	1.406 (2)
C3—H3	0.9500	C13—H13	0.9500
C4—C5	1.390 (2)	C14—C15	1.380 (2)
C4—H4	0.9500	C14—H14	0.9500
C5—C6	1.385 (2)	C15—C16	1.388 (2)
C5—H5	0.9500	C15—H15	0.9500
C6—H6	0.9500		
			
O1—S1—O2	118.23 (6)	S1—C7—H7B	110.1
O1—S1—C1	109.21 (6)	H7A—C7—H7B	108.4
O2—S1—C1	109.08 (6)	O3—C8—C9	122.11 (13)
O1—S1—C7	108.87 (6)	O3—C8—C7	121.11 (13)
O2—S1—C7	106.87 (6)	C9—C8—C7	116.75 (12)
C1—S1—C7	103.59 (7)	C10—C9—O4	111.88 (12)
C16—O4—C9	105.58 (11)	C10—C9—C8	132.54 (13)
C2—C1—C6	122.09 (13)	O4—C9—C8	115.52 (12)
C2—C1—S1	118.46 (11)	C9—C10—C11	106.31 (13)
C6—C1—S1	119.40 (11)	C9—C10—H10	126.8
C1—C2—C3	118.55 (13)	C11—C10—H10	126.8
C1—C2—H2	120.7	C16—C11—C12	118.92 (13)
C3—C2—H2	120.7	C16—C11—C10	105.45 (13)
C4—C3—C2	120.04 (14)	C12—C11—C10	135.62 (14)
C4—C3—H3	120.0	C13—C12—C11	117.97 (15)
C2—C3—H3	120.0	C13—C12—H12	121.0
C5—C4—C3	120.60 (14)	C11—C12—H12	121.0
C5—C4—H4	119.7	C12—C13—C14	121.27 (15)
C3—C4—H4	119.7	C12—C13—H13	119.4
C4—C5—C6	120.23 (14)	C14—C13—H13	119.4
C4—C5—H5	119.9	C15—C14—C13	122.05 (14)
C6—C5—H5	119.9	C15—C14—H14	119.0
C5—C6—C1	118.49 (13)	C13—C14—H14	119.0
C5—C6—H6	120.8	C14—C15—C16	115.62 (14)
C1—C6—H6	120.8	C14—C15—H15	122.2
C8—C7—S1	107.86 (10)	C16—C15—H15	122.2
C8—C7—H7A	110.1	O4—C16—C15	125.07 (13)
S1—C7—H7A	110.1	O4—C16—C11	110.78 (12)
C8—C7—H7B	110.1	C15—C16—C11	124.14 (14)
			
O1—S1—C1—C2	29.99 (13)	O3—C8—C9—C10	176.92 (15)
O2—S1—C1—C2	160.57 (11)	C7—C8—C9—C10	−1.0 (2)
C7—S1—C1—C2	−85.89 (12)	O3—C8—C9—O4	−0.1 (2)
O1—S1—C1—C6	−152.63 (12)	C7—C8—C9—O4	−178.03 (11)
O2—S1—C1—C6	−22.06 (14)	O4—C9—C10—C11	0.70 (16)
C7—S1—C1—C6	91.48 (13)	C8—C9—C10—C11	−176.42 (15)
C6—C1—C2—C3	−0.3 (2)	C9—C10—C11—C16	−0.87 (16)
S1—C1—C2—C3	177.00 (11)	C9—C10—C11—C12	177.66 (16)
C1—C2—C3—C4	−0.2 (2)	C16—C11—C12—C13	0.3 (2)
C2—C3—C4—C5	0.5 (2)	C10—C11—C12—C13	−178.10 (16)
C3—C4—C5—C6	−0.3 (3)	C11—C12—C13—C14	−1.1 (2)
C4—C5—C6—C1	−0.2 (2)	C12—C13—C14—C15	0.8 (2)
C2—C1—C6—C5	0.5 (2)	C13—C14—C15—C16	0.4 (2)
S1—C1—C6—C5	−176.78 (12)	C9—O4—C16—C15	−179.32 (14)
O1—S1—C7—C8	84.82 (11)	C9—O4—C16—C11	−0.36 (15)
O2—S1—C7—C8	−43.93 (11)	C14—C15—C16—O4	177.59 (13)
C1—S1—C7—C8	−159.05 (10)	C14—C15—C16—C11	−1.2 (2)
S1—C7—C8—O3	−67.61 (16)	C12—C11—C16—O4	−178.05 (13)
S1—C7—C8—C9	110.33 (12)	C10—C11—C16—O4	0.77 (16)
C16—O4—C9—C10	−0.23 (15)	C12—C11—C16—C15	0.9 (2)
C16—O4—C9—C8	177.42 (12)	C10—C11—C16—C15	179.74 (14)

### Hydrogen-bond geometry (Å, °)

**Table:**

*D*—H···*A*	*D*—H	H···*A*	*D*···*A*	*D*—H···*A*
C3—H3···O3^i^	0.95	2.55	3.1808 (19)	124
C7—H7a···O2^ii^	0.99	2.57	3.5383 (17)	165
C7—H7b···O1^i^	0.99	2.47	3.3746 (17)	152
C15—H15···O3^iii^	0.95	2.47	3.2742 (17)	142

Symmetry codes: (i) −*x*+3/2, *y*+1/2, −*z*+3/2; (ii) *x*, *y*+1, *z*; (iii) −*x*+1, −*y*, −*z*+1.
